# Aggregating Data for Computational Toxicology Applications: The U.S. Environmental Protection Agency (EPA) Aggregated Computational Toxicology Resource (ACToR) System

**DOI:** 10.3390/ijms13021805

**Published:** 2012-02-09

**Authors:** Richard S. Judson, Matthew T. Martin, Peter Egeghy, Sumit Gangwal, David M. Reif, Parth Kothiya, Maritja Wolf, Tommy Cathey, Thomas Transue, Doris Smith, James Vail, Alicia Frame, Shad Mosher, Elaine A. Cohen Hubal, Ann M. Richard

**Affiliations:** 1 U.S. EPA, National Center for Computational Toxicology, Research Triangle Park, NC 27709, USA; E-Mails: martin.matt@epa.gov (M.T.M.); sumitgangwal@gmail.com (S.G.); reif.david@epa.gov (D.M.R.); kothiya.parth@epa.gov (P.K.); smith.doris@epa.gov (D.S.); vail.james@epa.gov (J.V.); frame.alicia@epa.gov (A.F.); mosher.shad@epa.gov (S.M.); hubal.elaine@epa.gov (E.A.C.H.); richard.ann@epa.gov (A.M.R.); 2 U.S. EPA, National Exposure Research Laboratory, Research Triangle Park, NC 27709, USA; E-Mail: egeghy.peter@epa.gov; 3 Lockheed Martin, Research Triangle Park, NC, USA; E-Mails: maritja.a.wolf@lmco.com (M.W.); thomas.r.transue@lmco.com (T.C.) tommy.cathey@lmco.com (T.T.)

**Keywords:** computational toxicology, database, hazard, exposure, high-throughput screening, ACToR, ToxCastDB, ExpoCastDB, ToxRefDB, DSSTox

## Abstract

Computational toxicology combines data from high-throughput test methods, chemical structure analyses and other biological domains (e.g., genes, proteins, cells, tissues) with the goals of predicting and understanding the underlying mechanistic causes of chemical toxicity and for predicting toxicity of new chemicals and products. A key feature of such approaches is their reliance on knowledge extracted from large collections of data and data sets in computable formats. The U.S. Environmental Protection Agency (EPA) has developed a large data resource called ACToR (Aggregated Computational Toxicology Resource) to support these data-intensive efforts. ACToR comprises four main repositories: core ACToR (chemical identifiers and structures, and summary data on hazard, exposure, use, and other domains), ToxRefDB (Toxicity Reference Database, a compilation of detailed *in vivo* toxicity data from guideline studies), ExpoCastDB (detailed human exposure data from observational studies of selected chemicals), and ToxCastDB (data from high-throughput screening programs, including links to underlying biological information related to genes and pathways). The EPA DSSTox (Distributed Structure-Searchable Toxicity) program provides expert-reviewed chemical structures and associated information for these and other high-interest public inventories. Overall, the ACToR system contains information on about 400,000 chemicals from 1100 different sources. The entire system is built using open source tools and is freely available to download. This review describes the organization of the data repository and provides selected examples of use cases.

## 1. Introduction

Historically, information related to the effects of environmental chemicals has been widely distributed across numerous databases and sources. The task of consolidating these data resources was complicated by the diversity of non-standardized systems developed over the past 40 years of toxicology studies, ranging from online databases to compilations of individual electronic (and sometimes paper) documents. Previously, gathering all relevant information on a chemical required the search of tens to hundreds of sources and then manual compilation of the resulting data. To address this issue, ACToR (Aggregated Computational Toxicology Resource) was developed as a consolidated, searchable (by CASRN, name, chemical structure) collection of data on environmental chemicals. ACToR is available via the Internet and includes chemical identifiers and structures, physicochemical values*, in vitro* assay data and *in vivo* toxicology data, and source information and link-outs. Chemicals include but are not limited to those of interest to environmental scientists and regulators. In addition to information on environmental chemicals, data on pharmaceutical compounds is included because of the interaction between environmental and pharmaceutical toxicologists, both of whom are developing new methods for predicting human toxicity from *in vitro* and computational approaches. These approaches are heavily dependent on extensive data sets for building and validating models.

ACToR is currently being used to address three major goals: (1) making information on the health effects and exposure potential for environmental chemicals readily accessible; (2) characterizing gaps in knowledge of the toxicology of environmental chemicals; and (3) providing a resource for model-building to fill data gaps in environmental health risk information. ACToR has brought together data from sources whose identity and location are not widely known, or where data was not readily accessible in searchable or computable form. Of particular note are data from animal studies of pesticides that were previously publicly unavailable [1]. In addition, little toxicology information is available for many environmental chemicals, creating an environmental chemical “data gap” [2]. ACToR has been used to carry out a characterization of the data gap by compiling toxicology data on major classes of environmental chemicals, including those considered high and medium production volume chemicals, or those produced at more than 25K pounds/year, as defined under the Toxic Substances Control Act. From this analysis, we found that only about 25% of the most widely used environmental chemicals have significant toxicology data [3]. Computational toxicology, which uses a combination of *in vitro* data, chemical information, and computer modeling to predict chemical toxicity, represents a significant new approach to predicting human health risk for environmental chemicals. The U.S. Environmental Protection Agency (EPA) ToxCast screening and prioritization program [4] is a major effort in this area and was the driver for the development of ACToR. Descriptions of the use of ACToR in ToxCast are available elsewhere [3,5].

## 2. The Databases

### 2.1. Overall Organization

The ACToR system is comprised of four large interacting databases that share tables describing chemical identity and structure, but that maintain separate schemas for managing domain-dependent data on the chemicals. All of the databases are implemented in MySQL (http://www.mysql.com/), a freely available database system that can run on multiple platforms, including Linux, Windows and Mac. The MySQL databases can be freely downloaded so that other groups can develop custom data-mining applications using our data. In order to make the databases as portable as possible, we have largely used default database settings with the MyISAM database engine. The applications have acceptable performance despite using no significant tuning and containing tens of millions of data points.

**Figure 1:**
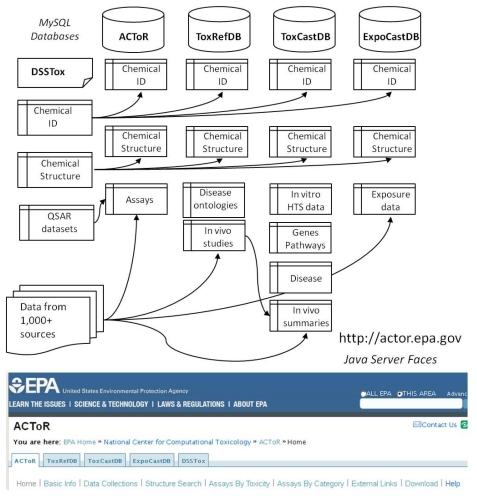
Organization of the databases. ACToR and the affiliated EPA databases shown use the same chemical identity and structure tables, fed by the DSSTox project (at left). Content from external data sources are fed into the ACToR database after filtering and formatting (bottom), with structures provided by PubChem if not available in DSSTox.

Figure 1 schematically illustrates the overall organization of the databases and their link to the DSSTox system. We have largely adopted the PubChem [6] model for describing chemicals, using the concepts of source-dependent “substance” and source-independent “compound”. A substance can be thought of as the chemical in the bottle, or physical sample that is actually tested A substance can have various identifiers, including one or more names, a Chemical Abstracts Services Registry Number (CASRN), a supplier, a purity value, *etc*., and is usually uniquely associated with a source. Each chemical may have numerous substance records. For example, there are many individual benzene substance records in the database, one for each experiment (or database) that produced (or reported) data on benzene. In contrast, there will be only one compound record, or structure representation in the database. A compound is the idealized representation of the structure of the chemical, which is the same for all of the substances to which it is linked. Hence, there will be one compound record in the database for benzene and all of the individual source-specific substance records will be linked to it.

Though the PubChem model was used for several aspects of ACToR, there are differences. PubChem is a public, user-depositor, structure-centric database whose primary mission is to store and aggregate bioassay data associated with chemical substances at the compound (or structure) level. In contrast, ACToR is concerned with aggregating publicly available data for a broader set of chemicals, including formulations, defined mixtures and complex mixtures. The latter includes ill-defined substances such as milk, mica, walnut shells, and molasses that are incorporated into commercial and industrial products and must be captured in the database despite having no well-defined structure. Therefore, we have explicitly added a layer termed “generic chemical”, which is typically defined by a CASRN, where available, and a preferred name. Additionally, if a chemical can be represented by a well-defined structure (e.g., benzene), it will have a compound (or structure) record linked to it and will additionally have one or more linked substance records. In contrast to PubChem, ACToR currently uses CASRN (or a generic chemical identifier when CASRN is unavailable), as a primary key since most of the toxicology and exposure literature and databases use this as a unique identifier. Use of CASRN (or the generic chemical identifier) allows us to aggregate over a broad range of data sources, even when chemical structures are not available.

As shown in Figure 1, each of the databases uses the same set of database tables describing substance, compounds and generic chemicals, carrying the main chemical IDs and chemical structure linkages. In practice, these tables are duplicated and periodically synchronized to allow the different databases to operate independently, and yet pull from the latest list of curated chemical structures.

The DSSTox project is coordinated with ACToR to ensure that the chemicals associated with the major EPA projects published within ACToR are correctly and consistently annotated by name, CASRN and chemical structure. Public data sources are replete with examples of incorrectly identified chemicals (incorrect or insufficiently precise names, incorrectly assigned CASRN and incomplete representations or explicit errors in structure). DSSTox has adopted strict information quality review standards and manually curates all of the chemical information assigned to substances used in the research efforts feeding the ToxRefDB, ToxCastDB and ExpoCastDB databases, in addition to producing QSAR-ready datasets for a variety of other public inventories pertaining to environmental toxicity.

Because data within ACToR is consolidated from hundreds of different sources, we have constructed several workflows that map raw data from individual sources into a small number of standard flat file formats. These files are then loaded into the appropriate database tables. Each new version of each of the databases is created by reloading all of the data into an empty set of tables. The data manipulation software is written in several languages, including Perl, Python, Java and R.

All of the described databases are accessible through the web at http://actor.epa.gov. Each of the main components can be accessed separately through tabs at the top of the page, and these interfaces allow browsing and searching capabilities. The current ACToR web site has limited search and modeling capabilities. However, since the data are fully downloadable in standard table format, users can readily employ a variety of public and in-house analysis tools. As will be described later, we are moving towards a more flexible, web-services architecture that will allow for the construction of a richer set of search and analysis utilities based on the back-end data.

Subsequent sections will provide more detail on the data organizational model and the major component databases within ACToR. The ACToR web site provides schema diagrams for each database, along with the ability to download some component databases or the complete ACToR database in MySQL format. Finally, the web interface code can be freely downloaded so that the complete ACToR system can be implemented locally.

### 2.2. ACToR

The goal of the core ACToR (Aggregated Computational Toxicology Resource) database and web site is to aggregate all publicly available information on chemicals in the environment, with a focus on information that pertains to toxicology and risk assessment. Given this lofty goal and the heterogeneity of “data”, we developed a simple and robust database schema to store information using a general concept of an “assay”. Essentially an assay is defined as a flat representation of data on a set of chemicals which can be organized into a table where the rows are chemicals and the columns can be any computed, tabulated, or measured attributes of the chemical. Employing this overarching definition of “assay”, each column is an “assay component” and each cell of the table is an “assay result”. In turn, each data source to which the assay results are linked is termed a “data collection”, which typically provides information that is then mapped to the substance, compound, and generic chemical and assay table entries. The “A” in ACToR indicates the aggregation of information at the level of the generic chemical, *i.e.*, from all substances and data collections mapping to a particular CASRN. Note that we include some substances without CASRN, and in those cases, a generic chemical identifier is assigned in place of the CASRN of the form “NOCAS_(…)”.

**Figure 2:**
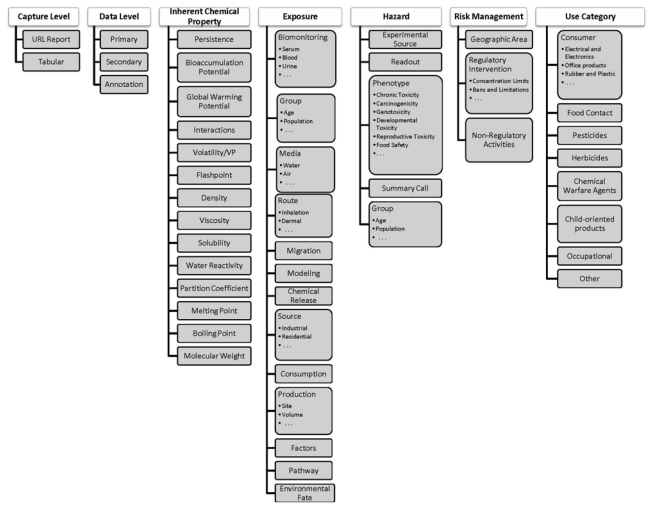
Illustration of assay categories used in ACToR. Assays (data sets) are put into one or more categories in this hierarchy to allow users to select only certain relevant types of data. This table shows the top level of this category hierarchy.

Because assays within ACToR are so heterogeneous, a further organization was required, which was implemented using a hierarchical set of assay categories. Figure 2 illustrates the basic assay taxonomy using the high-level concepts of Inherent Chemical Properties (ICP), Hazard, Exposure, Use Category, and Risk Management. These categories are used to label and organize the content of the data, whereas two other high level categories that pertain to the nature of the data capture and storage in ACToR (Capture Level and Data Level) are metadata concepts. Capture Level distinguishes cases where chemical data can be imported in tabular format from cases where ACToR simply stores a URL to a web-accessible data set or text report on the chemical. Data Level indicates whether the data is “primary” (*i.e.*, taken from the original source), “secondary” (*i.e.*, compiled from primary sources by others) or “annotation” (more descriptive information rather than data). An example of an “annotation” assay is a link to a Wikipedia article on the chemical that provides general descriptive information. It is important to stress that the data model used within ACToR is approximate, is not unique (*i.e.*, other organizational data models could be applied), is tailored to the categories of information sought for constructing toxicity risk assessments, and was built for the practical purposes of organizing and locating heterogeneous data to allow it to be meaningfully aggregated.

ICP describes properties that are inherent to the chemical and its structure, typically independent of biological target interactions, or that can be predicted using chemical structure models (QSARs). Examples of the former are molecular weight and boiling point, whereas examples of the latter are bioaccumulation potential or octanol water partition coefficient. “Hazard” largely describes data that are associated directly or indirectly with toxicology experiments. An example of the former would be a data collection of experimental results compiled from the literature such as provided by the Carcinogenic Potency Database [7] (a secondary source). An example of the latter would be an IRIS (Integrated Risk Information System) [8] or IARC (International Agency for Research on Cancer) [9] category or recommendation that considers a large body of experimental data in the literature. The experimental source may be an *in vivo* or *in vitro* experiment and the high-level phenotype being investigated may be carcinogenicity, developmental toxicity, *etc*.

Because of its central role in the study of chemical toxicity, we list below the complete hazard category hierarchy employed within ACToR:

- Hazard
- Hazard; Experimental Source
- Hazard; Experimental Source; *In Vitro*; (Biochemical, Cell Based)
- Hazard; Experimental Source; *In Vivo*; (Study Listing, Case Reports, Epidemiology)
- Hazard; Experimental Source; In Silico
- Hazard; Readout; (Genomics, HTS, HCS, Pathology)
- Hazard; Phenotype; (AcuteTox, Allergy, SubchronicTox, ChronicTox, Carcinogenicity, Genetox, Mutagenicity, DevTox, ReproTox, NeuroTox, DevNeuroTox, ImmunoTox, DermalTox, PhotoTox, HepatoTox, Endocrine, CardioTox, EcoTox, FoodSafe, ToxOther)
- Hazard; Summary Call; (MOA, ToxGuides)
- Hazard; Group; Age; (Fetus, Child, Juvenile, Adult)
- Hazard; Group; (Individual, Species)

Exposure categories are employed to facilitate consolidation of key exposure metrics. These include data describing exposure sources (industrial releases, consumer product use, *etc*.), environmental fate, chemical levels in environmental and biological media (indoor air, soil, drinking water, urine, serum, *etc*.), and exposure route (inhalation, dermal, absorption, ingestion). Use categories are employed to indicate how the chemicals are typically used within industry and the commercial realm (e.g., pesticides, pharmaceuticals, surfactants), which is useful information for exposure models. Finally, risk management categories carry information that pertains to expert judgment-based evaluation and perceptions of risk, such as derived safe exposure limits, thresholds of toxicological concern, *etc*.

Given the broadly diverse and overlapping nature of the data resources within ACToR, a given data set or assay may fall into multiple categories. For example, the EPA IRIS assessments, which review a large amount of data to provide route-specific reference doses (e.g., oral, inhalation) or weight-of-evidence calls (carcinogenicity), are categorized as follows:

- Capture Level; (Tabular and URL Report)
- Data Level; Secondary; Peer Reviewed
- Hazard; Experimental Source; *In Vivo*
- Hazard; Group; Human
- Hazard; Phenotype; (AcuteTox, Carcinogenicity, ChronicTox, …)
- Risk Management; Limit; Environmental

Data sources in ACToR include the U.S. EPA, Centers for Disease Control (CDC), U.S. Food and Drug Administration (FDA), National Institutes of Health (NIH), State agencies, corresponding government agencies in Canada, Europe and Japan, universities, the World Health Organization (WHO) and non-governmental organizations (NGOs). We have incorporated all chemicals and related assay data from PubChem for which CASRN could be extracted. The December 2011 release of the database contains information on approximately 1.7M substances, 280K chemical structures, 400K generic chemicals, 2730 assays and 16M individual data points from 1101 data collections.

**Figure 3:**
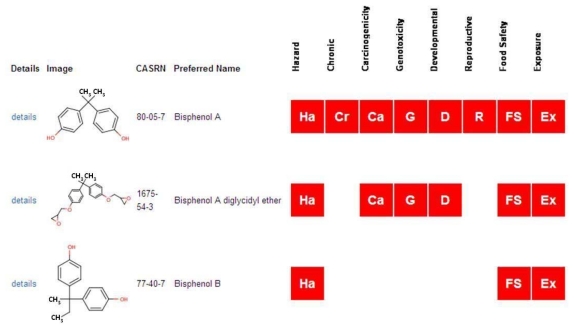
Screen shot from the ACToR web site for the high level view of data available for a set of chemicals. The view shows the chemical structure, name and CASRN, plus an indication of the types of data available for the chemical. In particular, we call out key phenotype categories (See Figure 2) and exposure. A red box in a column indicates that the database contains data for that chemical-category combination, and not that (for instance) the chemical causes cancer.

The web-accessible version of ACToR provides several methods to browse or search for data. The most common approach is to search for data on a single chemical by name or CASRN through a search box on the main page. Alternatively, a chemical structure search can be submitted using integrated tools from ChemAxon [10], where the search converts a drawn structure to SMILES and searches against SMILES representations of compounds that have been normalized using OpenBabel (http://openbabel.org/). A successful search will return one or more generic chemicals with assigned structures, with an indication of whether ACToR has information on the chemical for several key hazard phenotypes and exposure. Figure 3 shows a screenshot of the first few chemicals that are returned for the query “butyldithiocarbamate”. The second chemical on the list has some data on all of the key phenotypes and exposure, whereas the last one on the list only has some generic hazard data. It is important to note that a red box in this view simply indicates that data linking the chemical and an assay associated with carcinogenicity, *etc*. is available, not that the chemical is a carcinogen.

**Figure 4:**
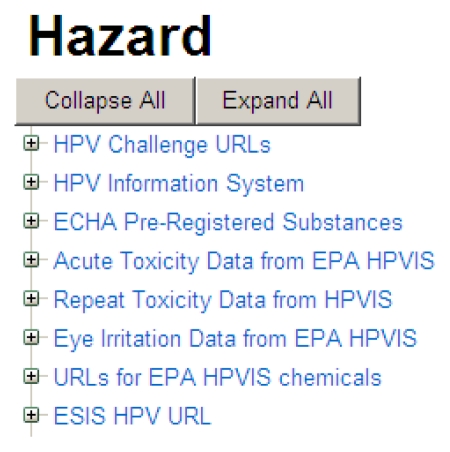
Screen shot of a portion of the ACToR data for Zinc dibutyldithiocarbamate

By selecting the “details” link to the left of the chemical structure, a user is presented with headers leading to a listing of the full data captured within ACToR. As an example, a portion of the data for Zinc dibutyldithiocarbamate is shown in Figure 4. The top section lists some of the overall Hazard data sets including data from the EPA, the EU, Health Canada and the National Library of Medicine. By selecting the “+” sign next to a data set, a user can access successive layers of detail pertaining to actual data and/or URLs linking to the data source.

In addition to searching by chemical, one can select links on the main ACToR page to view all of the data collections and can browse assays by the type of toxicity phenotypes or other categories to which they are linked.

### 2.3. ToxRefDB

ToxRefDB (Toxicity Reference Database) aims to capture traditional animal toxicity studies across a variety of study types and endpoints, including short-term and long-term systemic toxicity, cancer, reproductive toxicity, and developmental toxicity [1,11,12]. The ToxRefDB project initially focused on capturing previously unpublished high quality regulatory guideline studies required for chemical safety evaluation by the EPA. The study submissions were reviewed by the EPA’s Office of Pesticide Programs (OPP) and results consolidated into Data Evaluations Records (DER), which are the primary data source for ToxRefDB. Study results from these DER, as well as other high quality publically available studies, have been manually curated into ToxRefDB’s relational database model. The relational data database for ToxRefDB ensures data integrity by forcing specific vocabulary is used across all major ToxRefDB fields. The ToxRefDB relational format follows the following logic: a chemical can have many studies performed, each study can have multiple treatment groups (male and female, low-, mid-, and high-dose), and each treatment group can observe many effects. ToxRefDB has subsequently been integrated into the ACToR system, primarily through generic chemical linkages (*i.e.*, CASRN) and is available as a searchable database (http://actor.epa.gov/toxrefdb). ToxRefDB was designed to capture detailed study design, dosing, and treatment-related effect information. In addition to the relational design of the database, controlled and standardized vocabularies were used for the vast majority of fields to ensure the uniformity of the manually curated and entered legacy toxicity information. The current publically released version of ToxRefDB has study and chemical effect information on 474 chemicals, primarily pesticides due to their consistent and large data coverage of chronic, cancer, reproductive and developmental studies. The “Basic Info” page on the ToxRefDB website contains summary information about the database and the associated manuscripts. Importantly, the manuscripts release supplemental files with aggregated and detailed endpoints across the full ToxRefDB chemical library. These “flattened” endpoint files (*i.e.*, flat tabular listings) have been directly incorporated into the ToxCastDB system for predictive modeling exercises. The “Basic Info” page also provides information on the current database and chemical coverage counts for each study type (Table 1).

**Figure 5:**
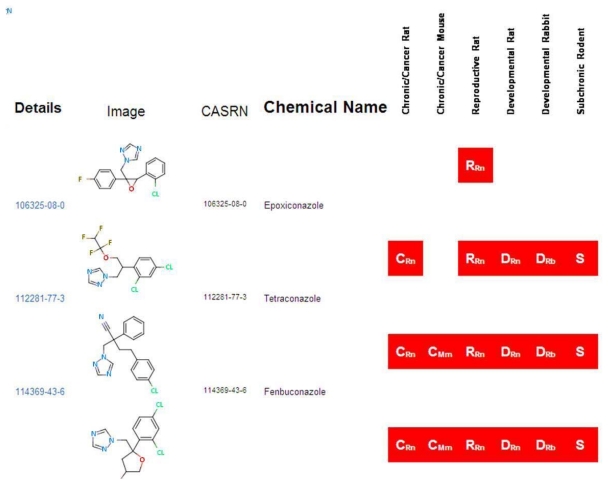
Screen shot from the ToxRefDB web site for the high level view of data available for a set of chemicals across the various available study types. This view is similar to the summary for ACToR (Figure 3), except that it indicates they standard types of studies for which the chemical has data in ToxRefDB.

The “Home” page of ToxRefDB, similar to that of all ACToR system databases, allows the user to search by generic chemical. As an example, the key word “azole” was used to search all 474 chemicals in ToxRefDB, by both their assigned chemical name and all synonyms, and resulted in the return of 46 chemicals (Figure 5). The red boxes indicate whether or not a study is available in ToxRefDB for the particular study type. A ”Generic Chemical Page” is displayed, as shown in the ACToR website; however, when accessing the ToxRefDB portion of ACToR, only chemicals with traditional toxicity data captured in ToxRefDB can be viewed. Under the “Toxicology Data” heading, all ToxRefDB data is displayed in a three-tiered structure. The first tier contains the study design information, including data quality, species and strain, dose administration, study type and citation information. The second tier contains treatment group and dosing information, while the third tier indicates the treatment-related effects observed at the various dose levels. The study information is available for viewing, but, due to the amount of detailed information stored within each tier, the system does not currently allow for detailed filtering of the data. However, a full download of the ToxRefDB data is available for each chemical as a csv file, enabling further analysis and viewing options.

**Figure 6:**
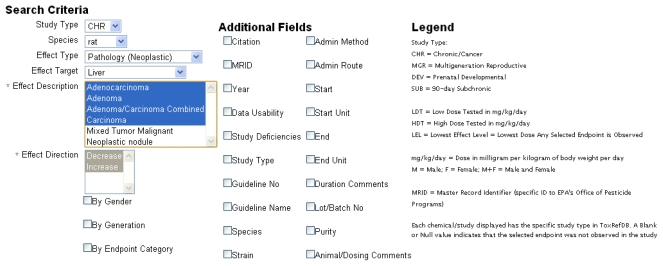
Screen shot from the ToxRefDB web site of the Endpoint search page with the search criteria and additional field information to be included.

**Figure 7:**
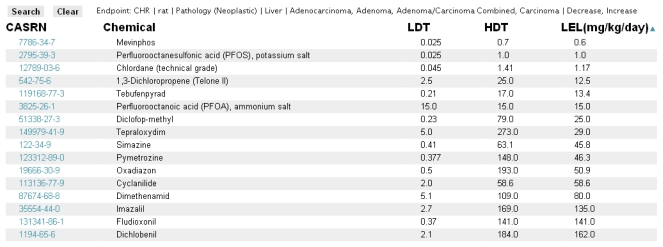
Screen shot from the ToxRefDB web site of the Endpoint search page with the results of the search displayed.

The primary search tool currently available within the ToxRefDB system is located in the “Search By Endpoint” tab. The page allows the user to select, from the standardized effect vocabulary, the exact search criteria of interest as well as the additional field information to be displayed (Figure 6). The results of searching, for example “Chronic/Cancer Rat Liver Neoplastic Pathology” returns the lowest effect level (LEL) in mg/kg/day dose that represents the lowest dose at which a treatment-related change in the selected effect or effects was observed (Figure 7). Each row from the returned search represents a unique study in ToxRefDB, with the low and high dose tested (LDT and HDT) provided for reference. If multiple effects are selected, a single LEL is returned which aggregates all selected effects with a primary goal of providing the field of predictive toxicology a tool for rapidly defining endpoints across a large chemical library. The endpoint search tool can also be used for researchers interested in delineating a set of reference chemicals with positive and negative outcomes for a particular effect or *in vivo* endpoint.

### 2.4. ToxCastDB

ToxCast is an EPA program that is generating high-throughput screening (HTS) data on thousands of chemicals across hundreds of *in vitro* assays [4,13]. The goal of the program is to use computational approaches to build predictive models of *in vivo* toxicity using *in vitro* HTS data and associated chemical and biological data. ToxCastDB was designed to capture all the data from ToxCast, related data generation efforts, and associated annotations. The upper level annotation and organization of summary *in vitro* HTS assay data within ToxCastDB is common to all other ACToR databases, *i.e.*, these data fit well into the basic ACToR assay table structure. However, because the ToxCast HTS assays are all run in concentration-response mode, ToxCastDB must additionally capture these individual data points. DSSTox is providing structure annotations and chemical sample details for ToxCast; hence, a MySQL version of the ToxCast DSSTox data is included here. Chemical sample details include testing-related information pertaining to source, lot/batch, supplier-reported purity and summary analytical QC data.

ToxCastDB also includes annotation data that link ToxCast HTS assays to genes, pathways and diseases. Most of the ToxCast HTS assays test for the interaction of chemicals with specific protein targets or assess the effects of chemicals on RNA or protein expression levels, and this knowledge is used to establish linkages with genes (*i.e.*, what gene or associated protein does the assay measure chemical effects on), most of which are of human origin. ToxCastDB incorporates basic gene information (Entrez gene id, symbol and name), and pathway-to-gene mapping from KEGG [14], WikiPathways [15], Ingenuity Pathway Systems (Ingenuity Systems, Inc, Redwood City CA), Pathway Commons [16] and REACTOME [17]. Chemical-gene information from the Comparative Toxicogenomics Database (CTD [18,19]) and gene-disease information from Online Mendelian Inheritance of Man (OMIM [20]) is also integrated.

The current web interface for ToxCastDB is found at http://actor.epa.gov/actor/faces/ToxCastDB/Home.jsp. This is a simplified version of the main ACToR web site that includes a search by name and CASRN, browsing by data collection and chemicals, and a view giving the links between assays and genes. The chemical-specific page shows the quantitative AC50 data (concentration at 50% of maximum activity) for those assays in which the chemical was positive. Also included are summary LEL values (lowest effective level) from ToxRefDB, which are the *in vivo* doses (analogous to an *in vitro* LEC, or lowest effective concentration) at which specified phenotypes were observed in animal experiments for the same chemical. The combination of *in vitro* and *in vivo* data across chemical sets is being used to develop predictive models of toxicity, described in the application section below.

The current version of ToxCastDB (toxminer_v17) contains data on the 309 ToxCast Phase I chemicals, tested in 594 *in vitro* assay endpoints (across 9 diverse assay technology platforms). The database is to be regularly updated with new data from subsequent phases, as well as data from several related projects using ToxCast assays. In order to handle this growth, a formal workflow has been developed to handle the immense volume of data generated across the diverse suite of ToxCast assays. This workflow uses collections of scripts to prepare, analyze, and consolidate data into standardized results for ACToR dissemination and use in modeling efforts. The formalization of this process into a sustainable workflow means that ToxCast results can be reliably reproduced from raw data as “best practices” for analysis evolve with new developments in related fields.

**Figure 8:**
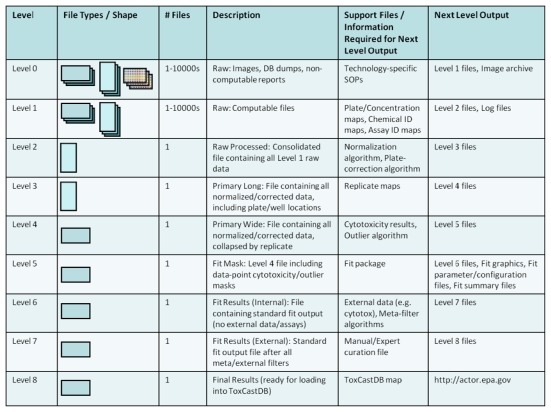
Data levels and processing steps in the ToxCast data workflow.

Figure 8 shows the levels at which data are tracked and created as they move through the workflow. The various levels are linked by scripts written in the R language so that they can be run on any machine by any member of the workflow team. Scripts linking certain key levels also generate auxiliary support files for archival, QA, and accounting purposes that include information on chemicals tested, assays included, data-fit summaries, *etc*. Proceeding through the workflow levels in Figure 8, “Raw” data (Level 0 or Level 1) may be received in a variety of formats, depending on the assay platform. Custom parsing scripts consolidate incoming data into analysis-ready files by reshaping and linking the blinded solution IDs sent to assay providers with DSSTox chemical substance annotations. Next, analysis proceeds through normalization, plate-correction, outlier detection, curve-fitting (estimating AC50s), and post-filtering. The post-filtering includes automated algorithms that use meta-data from other assays, as well as formalized manual curation of results. The end result is a standardized set of output (graphics plus files) containing AC50 estimates, model parameters, and assay “hit” criteria (*i.e.*, overall positive or negative assay call) that are loaded into ToxCastDB.

Throughout this workflow process, feedback is incorporated so that individual data sets are optimally handled. The result is a modular workflow that uses common machinery with data-set-specific, tunable parameters. For example, certain assay platforms require plate-level corrections, normalization to batch-level controls, or lack a defined maximum assay response. In each of these cases, parameters are adjusted accordingly, and the resulting graphical, tabular and summary results are reevaluated. Results are not published to ToxCastDB until consensus is reached amongst the ToxCast team.

### 2.5. ExpoCastDB

A critical need for risk assessment is the development of robust analytical approaches that use human exposure data, product use information, and modeled human behavior (e.g., activity patterns to systematically prioritize chemicals based on potential for exposure. Exposure can be modulated by chemical properties, uses throughout the product lifecycle, and by individual and population characteristics (e.g., lifestage and culture). To meet these needs, the EPA has developed the ExpoCast program in collaboration with internal and external partners and other stakeholders [21]. The goal of this research initiative is to develop novel approaches and metrics to screen and evaluate chemicals based on potential for biologically-relevant human exposures; *i.e.*, exposures that can be associated with key events in a disease process. Combining information from ToxCast with information from

ExpoCast will support risk assessment and decision making for improved public health. The ExpoCast research program is fostering development of exposure science to: (1) inform chemical prioritization; (2) improve understanding of system response to chemical perturbations resulting from environmental exposures and how these translate to relevant biological changes at the individual and population levels; and (3) link information on potential toxicity of environmental contaminants to real-world health outcomes. An early focus of this research program is to improve public access to exposure information.

ExpoCastDB was developed to improve access to human exposure data from observational studies, including those funded by the EPA National Exposure Research Laboratory measuring potential exposure to environmental chemicals. Similar to the previously discussed repositories, ExpoCastDB is integrated into the ACToR system through generic chemical linkages and is available as a searchable database (http://actor.epa.gov/actor/faces/ExpoCastDB/Home.jsp). Controlled vocabularies are used to facilitate searching and analyses across datasets and to encourage standardized reporting of observational exposure information. ExpoCastDB provides a separate interface within ACToR to facilitate linkage of exposure measurement data with data on toxicity, environmental fate, chemical manufacturing and usage, ToxCast HTS results, *etc*. The ExpoCastDB conceptual data model is designed to capture key information for characterizing exposure, details of study design, and metadata associated with sample analysis.

ExpoCastDB consolidates measurements of chemicals of interest in environmental and biological media collected from homes and child care centers. Data currently include the amounts of these chemicals found in food, drinking water, air, dust, indoor surfaces and urine. The domains of data implemented in the database are listed on the “Basic Info” page of ExpoCastDB and include chemical concentration measure, sample, study, location, medium and subject. The current publically released version of ExpoCastDB includes data for 99 unique chemicals primarily consisting of active ingredients in pesticide products. Chemical concentrations measured in samples collected for three observational studies are included: the American Healthy Homes Survey (AHHS) [22], the First National Environmental Health Survey of Child Care Centers (CCC) [23], and the Children’s Total Exposure to Persistent Pesticides and Other Persistent Organic Pollutants (both CTEPP NC and CTEPP OH) [24] studies.

**Figure 9:**
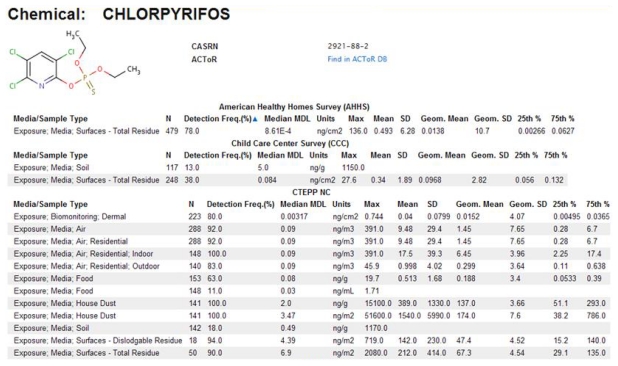
ExpoCastDB descriptive statistic chemical concentration results in different media for chloropyrifos.

In ExpoCastDB, chemical concentration data are organized by study, chemical and media type. General information about the individual studies as well as study-specific data can be accessed through the Study List page. Links are provided to descriptive statistics on chemical concentration (median method detection limit, max, mean, standard deviation, geometric mean, geometric standard deviation, and 25^th^ and 75^th^ percentile) for each of the chemicals in each medium sampled. The entire set of hierarchical data (extracted from the database) from each individual study is available for download from this page. Descriptive statistics can also be directly accessed for individual chemicals through the Chemical List page, or for individual exposure medium through the Media List page. For example, descriptive statistics for chloropyrifos concentrations in different media from all three studies can be accessed on the Chemical List page (Figure 9). The primary search tool currently available within the ExpoCastDB system is located on the Home page where information can be obtained by entering the chemical name or CASRN.

Future implementations of ExpoCastDB will include other data domains, such as laboratory methods used to detect a chemical, sampling method, sources, *etc*. Also, chemical exposure data from other studies will continue to be added to ExpoCastDB to improve access to extant exposure measurements. We plan to provide data visualization capabilities (e.g., scatter plots, probability plots, goodness-of-fit), and allow users to combine chemical concentration data from the same media across studies to obtain summary statistics and estimate distributional parameters. Further, exposure-related information on nanomaterials (e.g., particle number concentrations detected in air in occupational settings) [25] can be housed in ExpoCastDB as it becomes available.

### 2.6. DSSTox

DSSTox (Distributed Structure-Searchable Toxicity [26,27], http://www.epa.gov/ncct/dsstox) is a separate database effort from ACToR, focused on publishing expert curated,, standardized chemical structure-data files (SDF) associated with high-interest environmental toxicity data sets. A DSSTox structure-data inventory file consists of two parts: (1) DSSTox standard chemical information fields (including a molfile, SMILES, InChI, IUPAC names, molecular weight, Formula, name, CASRN, CID (compound ID) and GSID (generic substance ID); and (2) source-specific summary data fields contained within an individual DSSTox inventory (e.g., CPDBAS contains fields pertaining to tumor findings for rodent species for over 1500 generic substances from the Berkeley Carcinogenic Potency Database—CPDB [7]). DSSTox applies stringent quality review criteria to the accurate representation of chemical structures (including explicit treatment of stereochemistry, counter ions, complex forms, *etc*.) and test substances in association with toxicity data. In particular, careful attention is paid to ensuring accurate correspondence of chemical structure to CASRN and chemical name. General DSSTox quality review procedures are documented at http://www.epa.gov/ncct/dsstox/ChemicalInfQAProcedures.html and also in association with each published data file (in on-line documentation and downloadable log files). In the case of published chemical lists associated with toxicity data where original sample information (Source/lot/batch) is unavailable, such procedures focus primarily on ensuring consistency of various chemical identifiers (e.g., chemical name, CASRN and chemical structure) utilizing multiple public and commercial sources of information (e.g., ACD Labs Dictionary, National Library or Medicine’s ChemID Plus, CAS SciFinder). Chemical structures are confirmed by consensus of 3 trusted public sources, if possible (or CAS SciFinder, if the structure assignment to CAS is in question), and rendered in 2D format with accurate stereochemistry by an experienced PhD organic chemist curator. In the case of chemical inventories where original supplier documentation is published and publicly available (such as for ToxCast chemicals), review of Supplier documentation, Certificates of Analysis and Material Safety Data Sheets are consulted for more precise chemical identity verification (e.g., hydrate or salt form, CAS, purity, *etc*.).

The DSSTox Master chemical inventory—comprised of standard chemical information fields for all generic substances – currently contains in excess of 15K generic substances and 13K unique structures spanning more than 15 published inventories. The full DSSTox chemical inventory comprises an expert-curated subset of the full structure collection contained within ACToR. ACToR incorporates all published DSSTox inventories along with associated DSSTox Source content, including summary toxicity data associated with published studies or public databases (such as CPDB), where available. In addition, DSSTox provides both generic chemical structure annotation and chemical data management for each of the EPA project databases within ACToR. For ToxCast and Tox21 [28–30] programs, this includes tracking details of actual test samples (e.g., supplier, lot, batch, purity, analytical chemistry results, *etc*.), for more than 8000 chemicals. Given the high level of quality control applied to DSSTox content, ACToR considers DSSTox structural information in association with CASRN to be primary, overriding other potentially conflicting structural assignments from other public sources (e.g., PubChem). However, since DSSTox structures are unavailable for a large portion of ACToR’s overall data inventory (*i.e.*, compounds pertaining to many EPA and external public inventories), chemical structures from depositors to PubChem, in association with CASRN and name assignments, are used where available. Structures not available through DSSTox or ChemID vary considerably in accuracy within PubChem, based on the depositor source (not indicated in ACToR), so should be viewed as less reliable.

ACToR conveys the DSSTox origin of content in several ways. On each chemical data page, a chemical structure image is accompanied by an explicit Source label indicating “DSSTox”, where applicable. Similarly, DSSTox inventories (the full Master file, as well as sub-inventories) are labeled as “data collections” within ACToR (e.g., the name “NTP BSI Genetox Index” is listed under the Generic Chemical “Genetic Toxicity” heading, and clicking on “Details” is shown associated with the Data Collection: DSSTox NTPBSI). Finally, DSSTox Source-specific Record IDs (RIDs) for individual chemical records, which are uniquely assigned to the listing of unique generic substances (DSSTox_GSID) within every DSSTox inventory, are unique chemical identifiers for tracking and aggregating assay data within ToxCastDB. Both the DSSTox CID (structure/compound ID) and DSSTox RID are reported for ToxCast chemicals within ACToR (as Source Name CID and Source Name SID, respectively).

Although DSSTox content is fully incorporated within ACToR, the DSSTox project has a different primary focus of providing high quality chemical structure information and summary toxicity values in support of structure-based predictive toxicology. Hence, each public DSSTox inventory file (as well as the full DSSTox Master generic chemical information content) is published with extensive curation and documentation, and is publicly available for download from the DSSTox website in either SDF (mol file) or MS Excel formats. Finally, an important distinction of the ACToR and DSSTox databases is that the former aggregates on the basis of CASRN or generic substance ID, whereas the latter is primarily concerned with accurate chemical structural representation for a generic substance ID (or CASRN, where available). Hence, DSSTox does not publish chemical name synonyms or related (or discontinued) CASRN. ACToR aggregates information on the basis of CASRN, but where CASRN are mis-assigned or where different CASRN are assigned to closely-related compounds, the linkage of the CASRN to chemical structure within ACToR allows for effective chemical analog retrieval and data aggregation.

The DSSTox inventory is expanding to fill the needs of the growing EPA Computational Toxicology programs (ToxCast, ToxRefDB, Tox21, ExpoCast), as well as to register and standardize other high quality public structure inventories within and outside of EPA (e.g., EPA ECOTOX, and FDA CFSAN chemicals). Future goals will be to use DSSTox structure inventories and associated data to enhance the structure search and cheminformatics capabilities delivered by ACToR.

### 2.7. Curation

Data curation is an important activity for all of the databases. Some information on curation has been given in the individual sections, but here we summarize. For the core of ACToR, we largely accept data “as is” from the individual sources through spot checking. Since no data manipulations occur, the only potential errors happen during file reformatting. We do check CASRN and reject data for chemicals with invalid CASRN (*i.e.*, failing check digit verification). ToxRefDB uses extensive curation of the data because the input process involves manual extraction of quantitative data from text reports. QC steps include manual partial or complete reviews of data sets both inside the EPA and by chemical registrants. DSSTox hand curates all CASRN and chemical structures, applying expert review, consulting multiple public sources, and in cases where identity is in question, consulting the commercial CAS registry. As previously mentioned, the DSSTox project also curates the identity of all chemical samples used in the ToxCast program, which involves manual review of primary supplier documentation, such as Certificates of Analysis. ExpoCastDB currently takes quantitative values from source files and reformats, like ACToR. However a full automated check for data correctness against the source data is performed.

## 3. Applications

### 3.1. ACToR Application

**Figure 10:**
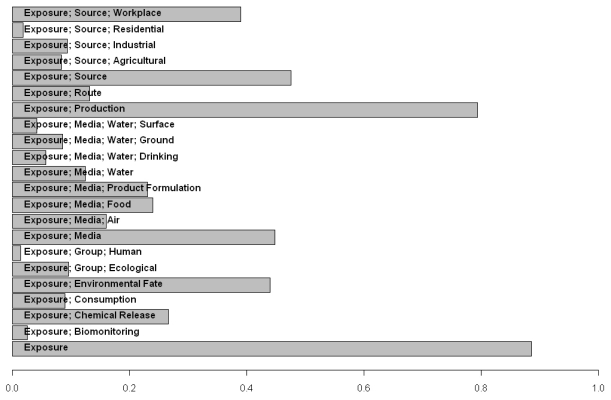
Summary of fraction of chemicals in the targeted inventory having exposure data by exposure category.

ACToR has been used to assess the data gaps for particular inventories of chemicals, determining the types of detailed analyses currently possible and aiding in the prioritizing of data generation moving forward. As an example of this type of application, ACToR was queried to locate the fraction of chemicals in a targeted inventory that had specified categories of exposure data from any source. The targeted inventory consisted of all chemicals (~8700 total chemicals) that were pesticide actives or inert ingredients, or potential water contaminants (the EPA Candidate Contaminant List (CCL) Universe). This particular target inventory is subject to the EPA Endocrine Disruptor Screening Program (EDSP) [31]. Figure 10 summarizes the results of the query in terms of the fraction of the inventory having any potentially relevant data. Approximately 40% of these chemicals have some workplace exposure data, whereas only a few have the corresponding residential exposure data. Production volume data is available for >80% of compounds in the target inventory, indicating that most of these are manufactured or imported in relatively large quantities [32].

### 3.2. ToxRefDB Application

ToxRefDB has been applied to multiple problem types, including retrospective and prospective questions. The application of ToxRefDB for prospective research efforts is discussed under the applications of ToxCastDB. Retrospectively, ToxRefDB has been used to assess the impact of specific traditional toxicity endpoints and parameters on the safety regulation of chemicals. For example, traditional toxicity testing for reproductive toxicity potential has relied heavily on the rat two-generation reproductive toxicity study. However, the importance of the second generation has come into question. An extended one-generation protocol has been proposed [33] that would only produce a second generation when triggered, would require far fewer animals, and would derive more toxicological and kinetic information from each animal used. To assess the impact of the second generation on risk assessment, ToxRefDB was used as the primary data source to systematically evaluate the question, relying on the highly standardized vocabulary and relational format of ToxRefDB. The results of the analysis indicate that the second generation does not greatly impact the interpretation of the reproductive study from a risk assessment perspective [34]. The two-generation retrospective analysis demonstrated the ability of ToxRefDB to provide a systematic review of traditional toxicity studies. Additional retrospective analyses are underway, including the relative impact and importance of running two species (both rat and rabbit) in prenatal developmental toxicity studies.

ToxRefDB also stores no-observed and lowest-observed adverse effect levels (NOAEL and LOAEL) for studies reviewed by EPA and used in the chemical registration process. Threshold of Toxicological Concern (TTC) [35] is an approach that uses NOAEL/LOAEL distributions and chemical structure characteristics to establish safe exposure levels for chemicals with limited to no toxicity information. ToxRefDB is currently being applied to TTC approaches in numerous venues, including assessing the applicability of the standard TTC to antimicrobial pesticide products and the refinement of TTC approaches for specific chemical classes. In the example of the antimicrobial TTC study, all available toxicity study information on antimicrobials is being collected and entered into ToxRefDB. Antimicrobial pesticides typically have less available toxicity data compared to conventional pesticides and, thus, underscore the need for alternative safety assessment approaches. With the full food-use antimicrobial traditional toxicology dataset available in a standardized and relational format, detailed analysis of the NOAEL/LOAEL distributions across study type, endpoint categories and structural classes can be obtained and compared to other TTC analyses. If found to be similar, then all or a portion of non-food-use antimicrobials could be evaluated using a TTC approach.

### 3.3. ToxCastDB Application

A major goal of the ToxCast project is to use a combination of *in vitro* and *in vivo* data, along with associated chemical, biological and mechanistic information, where available, to build predictive toxicology models. One approach starts by calculating statistical associations between *in vitro* targets (assays) and a particular toxicity *in vivo* endpoint. For example, the set of chemicals with associated rat liver tumor testing data from ToxRefDB was evaluated in relation to ToxCast HTS assay data for approximately 240 chemicals. Both the *in vivo* endpoint and the HTS assay results were first dichotomized, *i.e.*, summarized into two categories, *i.e.*, the chemical causes or does not cause liver tumors, and for each ToxCast assay, the chemical produces a “hit” (positive) or does not produce a hit (negative). From this array of binary results, *in vivo vs. in vitro* for a series of chemicals, an odds ratio can be calculated. For instance, chemicals that are androgen receptor antagonists as measured by an assay from the NIH Chemical Genomics Center, have a 5.4-fold higher odds of being rat liver carcinogens than chemicals that were negative in this assay (95% confidence interval is 1.9 to 15) Assays for which this odds ratio is computed to be large (*i.e.*, chemicals hitting this assay are found to have a much higher than chance odds of causing liver tumors) lead us to form hypotheses of the form: chemical perturbations of the gene or pathway probed by the assay increases the risk that the chemical will initiate or promote tumors. Next steps in the analysis workflow include building multivariate models that combine many individually predictive assays for the given endpoint, and forward validation of the model with chemicals not included in the initial training data set. Being able to access gene and pathway information directly linked to assay data from within the database enables a user to construct queries that can shed light on (for instance) whether assays linked to a particular endpoint probe a single key pathway or multiple pathways. Additionally, we can link directly to the literature on the predictive genes and pathways via Entrez URLs provided in the ToxCastDB web pages.

We have published examples of these analyses covering carcinogenicity and developmental reproductive toxicity. Judson *et al.* [13] used the simple univariate analysis described above to show that, within the ToxCast Phase I chemical set (which comprises mostly pesticide active ingredients), perturbations to several pathways were significantly associated with liver preneoplastic and neoplastic lesions. One target class includes the peroxisome proliferators-activated receptors (PPAR) alpha and gamma pathways. The link between PPAR signaling perturbations and liver tumors in rodents is well-documented, but our example shows how such associations can be independently “discovered” using this computational approach, providing a proof-of-principle. The approach, as such, can be characterized as “chemical epidemiology”. In a second example, Kleinstreuer *et al.* [36] have derived a signature (*i.e.*, an association of a portion of an *in vitro* profile to an *in vivo* response) to predict whether a chemical could be a vascular disruptor during early embryonic development. The consequence of this behavior would be to disrupt (among other things) limb development. This analysis once again compared the ToxCast Phase I *in vitro* data with data from ToxRefDB for prenatal development toxicity studies in rats and rabbits. The particular pathways found to be associated with vascular disruption included inflammatory chemokine signaling, the vascular endothelial growth factor pathway, and the plasminogen activating system, all of which have documented or clearly rationalized potential mechanistic ties to the endpoint under study. In a third example, Martin *et al.* [37] built a multi-variate model of reproductive toxicity in rodents. In this case, the endpoint is a composite of male and female effects on fertility and reproductive fitness. The resulting predictive model includes assay-related terms for PPARA and PPARG signaling, estrogen and androgen receptor activity, activity against the Pregnane-X receptor (PXR) and generalized activity against liver CYP450 enzymes and GPCRs. This quantitative classification model of an endpoint that has defied previous modeling attempts due to its biological and testing complexity and data scarcity, produced a significant balanced accuracy (average of sensitivity and specificity) in a cross-validation test of 74%. Finally, Sipes *et al.* [38] have built a model that predicts developmental toxicity endpoints such as cleft palate and urogenital defects in rat and rabbit. Chemical targets and corresponding assays associated with these endpoints include the retinoic acid receptor (RAR), interleukins IL1a and IL8, and the transforming growth factor beta (TGFβ). Furthermore, there is an interesting link between the cancer and the developmental ToxCast models. Expression of the chemokine CCL2 is known to be associated with vasculogenesis. In our models, we see increased CCL2 expression associated with cancer progression, while decreased expression is associated with a variety of developmental defects.

### 3.4. ExpoCastDB Application

ExpoCastDB is the latest EPA effort to address the decades-old call to increase access to human exposure data in order to support exposure modeling and advance public health through improved management of chemical risks [39,40]. Whereas considerable progress already has been achieved in making data from observational human exposure measurement studies (including datasets, study design documents, and metadata) available to the public through the Human Exposure Database System (HEDS, http://www.epa.gov/heds), it is the consolidation of exposure data into ACToR that allows for improved linkages with toxicity databases. ExpoCastDB can be used to obtain input distributions for probabilistic human exposure models and to answer a variety of questions about the occurrence of chemicals in the microenvironments in which humans exist. Probabilistic human exposure models are increasingly being used in the exposure and risk assessment process [41] but have tremendous input data requirements, including distributional parameters for the chemicals of interest in relevant exposure media. As described above, ExpoCastDB offers straightforward access to relevant distributional parameters (e.g., geometric mean and geometric standard deviation) in multiple exposure media for several commonly encountered chemicals, often from multiple studies.

The occurrence of chemicals within studies may be investigated with ExpoCastDB. For example, simple questions that can be addressed include: How frequently was a particular pesticide (e.g., esfenvalerate) detected in the nationwide AHHS study? Of the chemicals measured in CTEPP, which chemicals are found at the highest concentrations in indoor air? What is the distribution (or range) of malathion levels measured in wipe samples in CCC? Comparisons may also be made across studies. For example: How does the average permethrin loading differ between the homes in AHHS and the daycares in CCC? In addition, more challenging questions may be addressed by downloading the individual sample-level structured data, such as: What level of co-occurrence among environmental phenols (such as bisphenol A) would be expected in indoor environments based on data from CTEPP? The results obtained in response to questions such as the examples given above may then be used in conjunction with readily available algorithms [42] and exposure factors [43] to produce quick deterministic estimates of the uptake resulting from exposure to specific chemicals.

By providing the capability to mine data across multiple studies and a range of chemicals, ExpoCastDB is also intended to facilitate knowledge-driven hypothesis development. Currently, analysis is being conducted to relate chemical properties with the distribution of contaminants in different media as measured in the indoor environment. Efforts are currently being made to facilitate acquisition of exposure data, beginning with data from previous EPA studies. Efforts are also underway to acquire data from partners within the Federal government and among the larger exposure science community.

## 4. Future Directions and Conclusions

The ACToR system is a large and growing repository of data on chemicals in the environment. The main components of ACToR contain information on chemical identity and structure, *in vivo* toxicology experiments, *in vitro* screening, and exposure. We have described the structure of the databases, the types of data included and how it is organized and annotated, plus some specific applications.

A known limitation of ACToR, presently, is its primary reliance on CASRN for aggregation of chemical and assay information, since chemical structures are most often unavailable for many public inventories of interest. Hence, such data aggregation is limited to the particular chemical (or CASRN) of interest. However, improving both the quality and breadth of the chemical structure annotations within ACToR is viewed as a priority. With chemical structures comes the ability to aggregate information more broadly on the basis of chemical similarity using a variety of potentially useful metrics (e.g., toxicity structure alerts, reactivity features, calculated properties, structure fingerprints, *etc*.). Use of the concepts of chemical analogy and “read-across”, in turn, comprise an important strategy for addressing the problem of data gaps. Such similarity metrics are being developed and will be incorporated into future versions of ACToR to enable a user to view information across a chemical group or class.

ACToR currently has a large user community who access the data either through the web interfaces or by downloading all or parts of the database and building custom applications. A common and understandable criticism of the system is that it provides too much information, much of it not necessarily relevant to the task at hand. To address this issue, we are beginning a major new effort to create the infrastructure to build so-called “decision-support dashboards” or dashboards for short. A dashboard is conceptually simply a web page that provides a user or decision maker the data that they need to do the job at hand, and only that data. Because there are many different types of decisions, and a correspondingly large number of decision makers, one needs a flexible system to quickly construct custom dashboards. This effort will use web services that can take generic queries in the form of URLs, and then return the results as XML (Extended Markup Language). This can then be coupled with java-based toolkits, which allow rapid development of complete dashboards. An important aspect to the dashboards will be the assay categories described above. This will allow decision makers to (for instance) automatically only select data that is primary and peer-reviewed.

Another powerful approach we are beginning to incorporate into the ACToR system is knowledgebase tools. A knowledgebase is essentially a database plus an ontology that allows a rich and flexible description of entities and their relationships. This then allows for the use of all the tools developed as part of semantic web technology [44]. One promise of knowledgebase technologies is the ability to integrate heterogeneous data (and the ACToR databases are an extreme example of this) and to discover new facts or trends or connections.

Finally, we are working with other groups in the U.S., Europe and elsewhere to integrate ACToR with other similar databases. Any one database (or data warehouse) cannot hope to hold all relevant chemical or toxicology data, and different groups will design their database and user interfaces to meet variant use cases. Coordinating these databases or other sources of information will enhance all users’ searches. However, it is important in a world of limited resources to make sure that all of these public data resources make as much use of one another as possible. All these efforts contribute to the goal of ensuring maximal transparency and ease of public access to important data on the large number of chemicals in the environment.

## Figures and Tables

**Table 1:** Study and chemical counts from the ToxRefDB website.

Summary statistics	
Study Count	1978
Chemical Count	474
Combined Chronic/Cancer Rat	324
Combined Chronic/Cancer Mouse	324
Multigeneration Reproductive Rat	352
Prenatal Developmental Rat	365
Prenatal Developmental Rabbit	331
Subchronic Rodent	302

## Abbreviations

AC50: Concentration at 50% of maximum activity
ACToR: Aggregated Computational Toxicology Resource
AHHS: American Healthy Homes Survey
CASRN: Chemical Abstracts Registry Number
CCC: First National Environmental Health Survey of Child Care Centers
CCL: Candidate Contaminant List
CCL2: Chemokine (C-C motif) ligand 2
CDC: Centers for Disease Control
CFSAN: Center for Food Safety and Applied Nutrition
CID: Compound ID
CPDBAS: Carcinogenic Potency Database Summary
csv: comma-separated file
CTD: Comparative Toxicogenomics Database
CTEPP: Children’s Total Exposure to Persistent Pesticides and Other Persistent Organic Pollutants
CYP450: Cytochrome P450 enzyme
DER: Data Evaluation Record
DSSTox: Distributed Structure-Searchable Toxicity
EDSP: Endocrine Disruptor Screening Program
EPA: Environmental Protection Agency
ExpoCastDB: ExpoCast Database
FDA: U.S. Food and Drug Administration
GPCR: G-Protein Couple Receptor
GSID: Generic Substance ID
HCS: High-Content Screening
HDT: Highest Dose Tested
HEDS: Human Exposure Database System
HTS: High-Throughput Screening
IARC: International Agency for Research on Cancer
ICP: Inherent Chemical Property
IL1a: Interleukin 1a
IL8: Interleukin 8
IUPAC: International Union of Pure and Applied Chemistry
InChI: IUPAC International Chemical Identifier
IRIS: Integrated Risk Information System
KEGG: Kyoto Encyclopedia of Genes and Genomes
LDT: Lowest Dose Tested
LEL: Lowest Effect Level
LOAEL: Lowest Observed Adverse Effect Level
MOA: Mode of Action
NGO: Non-Governmental Organization
NIH: National Institutes of Health
NOAEL: No Observed Adverse Effect Level
OMIM: Online Mendelian Inheritance of Man
PPAR: Peroxisome Proliferators-Activated Receptor
PXR: Pregnane-X receptor
QSAR: Quantitative Structure Activity Relationship
RAR: Retinoic Acid Receptor
SDF: Structure Data File
SID: Substance ID
SMILES: Simplified Molecular Input Line Entry Specification
TGFβ: Transforming growth factor beta
TTC: Threshold of Toxicological Concern
ToxCastDB: ToxCast Database
ToxRefDB: Toxicology Reference Database
WHO: World Health Organization
XML: eXtended Markup Language
